# All-nitride Al_*x*_Ga_1−*x*_N:Mn/GaN distributed Bragg reflectors for the near-infrared

**DOI:** 10.1038/srep42697

**Published:** 2017-02-15

**Authors:** Giulia Capuzzo, Dmytro Kysylychyn, Rajdeep Adhikari, Tian Li, Bogdan Faina, Aitana Tarazaga Martín-Luengo, Alberta Bonanni

**Affiliations:** 1 Institut für Halbleiter-und-Festkörperphysik, Johannes Kepler University, Altenbergerstr. 69, A-4040 Linz, Austria; 2 Institute of Physics, Polish Academy of Science, al. Lotnikow 32/46, 02-668 Warsaw, Poland.

## Abstract

Since the technological breakthrough prompted by the inception of light emitting diodes based on III-nitrides, these material systems have emerged as strategic semiconductors not only for the lighting of the future, but also for the new generation of high-power electronic and spintronic devices. While III-nitride optoelectronics in the visible and ultraviolet spectral range is widely established, all-nitride efficient devices in the near-infrared (NIR) are still wanted. Here, through a comprehensive protocol of design, modeling, epitaxial growth and in-depth characterization, we develop Al_*x*_Ga_1−*x*_N:Mn/GaN NIR distributed Bragg reflectors and we show their efficiency in combination with GaN:(Mn,Mg) layers containing Mn-Mg_*k*_ complexes optically active in the near-infrared range of wavelengths.

Over two decades ago a series of fundamental breakthroughs in the area of gallium nitride (GaN)-based semiconductor materials has led to the first demonstration of high efficiency and high brightness blue light emitting diodes (LEDs)^1^,^2^. Currently, GaN-based blue and white LEDs reach efficiencies exceeding those of any conventional light source and III-nitride-based heterostructures represent the building-blocks not only of state-of-the-art laser diodes^3^, blue and white LEDs^4^, but also of high mobility transistors^5^,^6^, high power electronic^7^,^8^ and spintronic^9^ devices. The technological importance of III-nitrides is justified by a number of remarkable properties, including a widely tunable band-gap, the availability of both *n*- and *p*-type material, a sizable thermal stability and large heat conductivity. In order to extend the functionalities of III-nitride systems to the near-infrared (NIR) range – *e*.*g*. for telecommunication applications – currently these materials are either doped with rare earths and Er in particular^10^,^11^,^12^, or alloyed with a considerable amount of in refs 13,14, challenging the epitaxial growth and the homogeneity of the layers^15^.

Recently, we have reported that the co-doping of GaN with Mn and Mg results in the formation of robust cation complexes Mn-Mg_*k*_^16^,^17^, responsible for a room-temperature (RT) broad IR emission that covers two of the telecommunication windows, respectively centered at 1.33 *μ*m and 1.55 *μ*m, opening wide perspectives towards the realization of efficient NIR devices not requiring rare earths or In.

Moreover, by embedding in an optical cavity layers of GaN:(Mn,Mg) containing the Mn-Mg_*k*_ complexes, a variety of NIR opto-electronic devices, like vertical-cavity surface-emitting lasers (VCSELs)^18^,^19^, resonant-cavity light emitting diodes (RCLEDs)^20^, and single photon emitters (SPE)^21^,^22^, can be envisaged.

Distributed Bragg reflectors (DBR) are essential elements of an optical cavity and while stacks of dielectric materials deposited by electron beam evaporation are well established in the fabrication of DBRs for the NIR range^23^,^24^,^25^, the epitaxial growth of semiconductor-based DBRs by metalorganic vapor phase epitaxy (MOVPE) or by molecular-beam epitaxy (MBE)^26^,^27^ is highly desirable, since in this way the optically active layers can be grown directly on top of a buried DBR or sandwiched between two reflectors forming a resonator. Although these epitaxial protocols are widely reported for the ultraviolet (UV)^28^,^29^,^30^,^31^ and deep-UV^32^,^33^,^34^ range, the development of all-nitride NIR DBR/active region structures is in its infancy.

In a DBR the optical stop-band, *i*.*e*. the narrow range of wavelengths for which the propagation of light is strongly inhibited, is essentially due to multiple processes at the interfaces of a stack consisting of the repetition of two alternating layers – a Bragg pair – with respectively low and high refractive index. The separation between subsequent interfaces should be a multilple of a quarter of the design wavelength. The performance of the reflector is determined by: (i) the contrast in the refractive index between the two materials of the Bragg pair and (ii) by the number of pairs. Several groups reported on the fabrication of Al_*x*_Ga_1−*x*_N/GaN DBRs in the UV and visible range and in the majority of reports strain engineering comes into play, due to the necessity of overcoming the detrimental effects of stress relaxation originating from the lattice mismatch between GaN and its alloys. Among the procedures employed, we recall the use of GaN/Al_*x*_Ga_1−*x*_N or GaN/AlN superlattice (SL) insertion layers to reduce the biaxial tensile strain and to quench the generation of cracks^28^,^35^,^36^, useful also in the case of thick Al_*x*_Ga_1−*x*_N films^37^. Alternative solutions consist in inserting single or multiple AlN interlayers during the growth of the DBR sequence^38^, supported by an Al_*x*_Ga_1−*x*_N layer or buffer^28^,^39^,^40^.

An optional approach to fabricate and implement all-nitride DBRs efficient in the UV and visible range is to grow perfectly lattice matched Al_*x*_In_1−*x*_N/GaN or Al_*x*_In_1−*x*_N/Al_*y*_Ga_1−*y*_N superlattices using molecular beam epitaxy (MBE) or MOVPE^28^,^41^,^42^,^43^. Fabrication of Al_*x*_In_1−*x*_N/GaN DBR by MBE^19^,^28^,^44^ and by MOVPE^41^,^43^, functional in the UV and visible range, was achieved for Al concentrations ≤0.5 in Al_*x*_In_1−*x*_N. One major challenge is represented by the markedly different growth conditions required for the two materials. For instance^28^, in the MOVPE process Al_*x*_In_1−*x*_N with high In content grows in a N_2_ atmosphere at ~700 °C while Al_*y*_Ga_1−*y*_N and GaN are deposited in a H_2_ atmosphere at ~1000 °C. In order to achieve high crystallinity, stoichiometric Al_*x*_In_1−*x*_N layer extremely stable growth conditions are required. Growth and fabrication of Al_*x*_In_1−*x*_N based DBR in the NIR wavelength range would be even more challenging requiring higher number of superlattice periods in comparison with the UV case, implying stable growth conditions over much longer time scales.

We have recently demonstrated, that the incorporation of <1% of Mn during the epitaxy of Al_*x*_Ga_1−*x*_N, affects the plastic relaxation of the layers and increases substantially their critical thickness on GaN^45^.

In this work, we report on the design and fabrication of Al_*x*_Ga_1−*x*_N:Mn/GaN DBRs grown by MOVPE for the spectral region between 900 nm and 1500 nm and on their effect on the NIR emission from a GaN:(Mn,Mg) active layer.

## Results

The studied samples are epitaxially grown by MOVPE and include Al_*x*_Ga_1−*x*_N:Mn/GaN Bragg pairs, eventually overgrown with a GaN:(Mn,Mg) active layer. Details on the fabrication and characterization are provided in the Methods. Modeling of the reflectivity supported by the transfer matrix method (TMM)^46^ and spectroscopic ellipsometry measurements serve as basis for the design of the heterostructures, as highlighted in the Methods and in the Supplementary Information.

### Refractive indices and design

The refractive indices of Al_*x*_Ga_1−*x*_N:Mn alloys with different Al content and 0.2% of Mn are reported in Supplementary Fig. S1 together with those extrapolated from the model based on the first-order Sellmeier dispersion formula employed by Özgür *et al*.^47^ for Al_*x*_Ga_1−*x*_N. In order to obtain high reflectivity and a wide stop-band, a significant difference in the refractive indices of the two materials of the Bragg pairs is required, implying that the higher the concentration of Al in the Al_*x*_Ga_1−*x*_N layers, the more pronounced is the optical contrast with the GaN counterpart. However, one must take into consideration that the critical thickness of Al_*x*_Ga_1−*x*_N on GaN^48^ decreases dramatically with increasing the Al content *x* and the strain due to the lattice mismatch is released through the formation of dislocations and eventually through cracking of the structure. On the other hand, as already mentioned, the introduction of as less as 0.2% of Mn into Al_*x*_Ga_1−*x*_N allows us to increase significantly its critical thickness on GaN. By taking into account the limitations related to the epitaxial growth of mismatched materials – but taking advantage of the surfactant effect of Mn – and having GaN as high refractive index material and Al_*x*_Ga_1−*x*_N:Mn as low refractive index layer, we compromise on a target Al content *x* = 0.27. Moreover, we give for a stop-band in the wanted range – which includes, at 1200 nm, the most intense emission from the Mn-Mg_*k*_ complexes in the GaN:(Mn,Mg) active layer – a thickness of 137 nm for the Al_0.27_Ga_0.73_N:Mn layer and of 131 nm for the GaN one. With these values, we show that one can reach already a 62% of reflectance with a multilayer structure consisting of 20 strained Bragg pairs.

**Figure 1:**
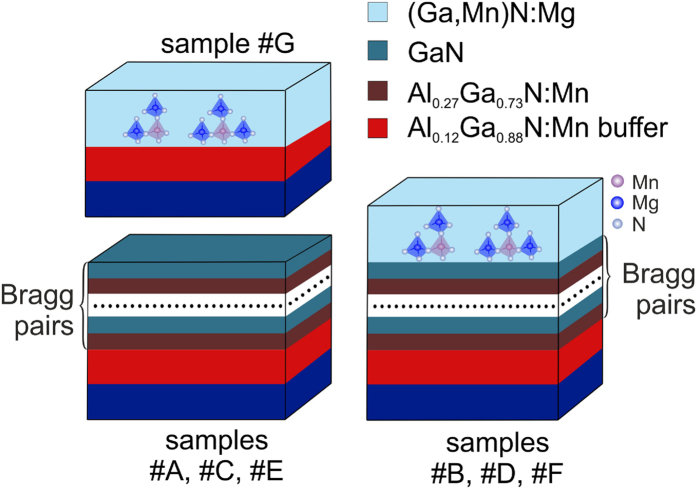
Sketches of the samples structures. Sample #G is a reference without DBR and consisting of a GaN:(Mn,Mg) active layer deposited directly on the Al_0.12_Ga_0.88_N:Mn buffer.

The schematic model of the studied structures is reported in Fig. 1, while the number of Bragg pairs for each investigated sample is provided in Table 1, together with details on the presence of the active layer.

### Towards an optimized DBR

**Figure 2:**
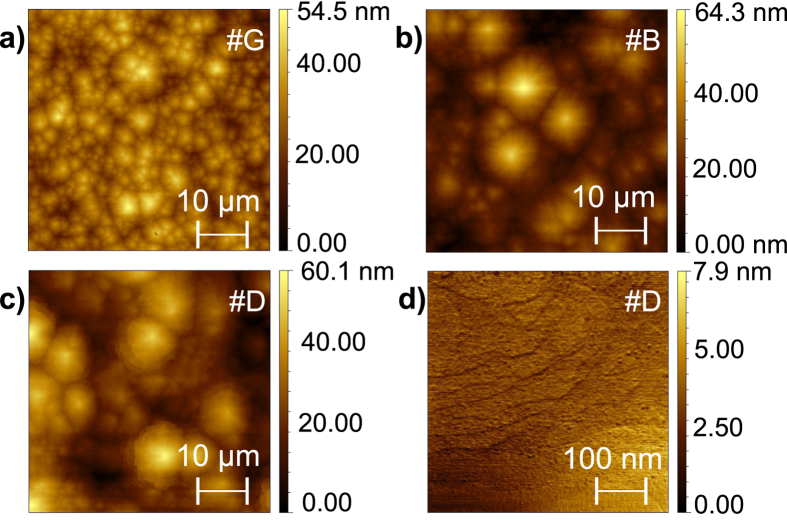
Atomic force micrographs for: (**a**) sample #G (reference without DBR), (**b**) sample #B (5-fold DBR and GaN:(Mn,Mg) active layer), (**c**,**d**) sample #D (10-fold DBR and GaN:(Mn,Mg) active layer). On the larger scale (**a**–**c**), the features typical of Mn-doped samples are visible, while in the 1 *μ*m-scale picture (**d**) atomic terrace edges characteristic of a step-flow growth mode can be distinguished.

A protocol of in-depth post-growth characterization of the structures is employed in order to establish the relation between growth parameters, crystallographic arrangement, chemical composition and optical response of the investigated structures. On the large scale, the surface of all the samples studied by atomic force microscopy (AFM) and reported in Fig. 2(a–c) shows a morphology already observed in the Al_*x*_Ga_1−*x*_N:Mn samples studied by our group recently^45^. In the presence of the GaN:(Mn,Mg) active layer and with increasing number of Bragg pairs, the average size (both in-plane and in the growth direction) of the surface features increases, as seen when comparing the reference sample #G (active layer directly deposited on the buffer) in Fig. 2(a) with Fig. 2(b,c), where a 5-fold and a 10-fold DBR have been added, respectively. In the high resolution images, on the other hand, it is possible to distinguish the atomic terrace edges characteristic of a step-flow growth mode, as evidenced in Fig. 2(d).

**Figure 3:**
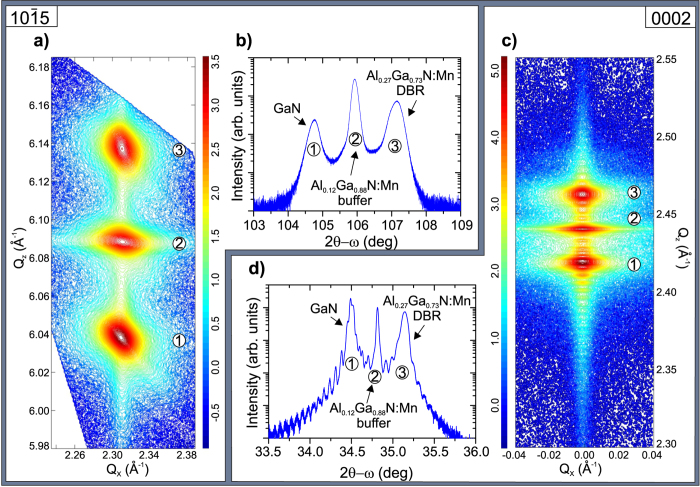
Panels (a,b) RSMs and a radial 2*θ*-*ω* scan around asymmetric GaN and Al_*x*_Ga_1−*x*_N(:Mn) reflections (10
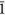
5). Panels (c,d) RSMs and a radial 2*θ*-*ω* scan around symmetric GaN and Al_*x*_Ga_1−*x*_N(:Mn) reflections (0002) with thickness fringes. The structure is grown pseudomorphically on the Al_0.12_Ga_0.88_N:Mn buffer. The intensity is reported in logarithmic scale. All scans refer to sample #D.

X-ray diffraction reciprocal space maps (RSMs) and radial 2*θ*-*ω* scans measured around the asymmetric (10
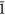
5) and symmetric (0002) reflections of GaN and Al_*x*_Ga_1−*x*_N(:Mn) – reported in Fig. 3(a–d) – show that all the structures under investigation are grown pseudomorphically. The Bragg reflections for the GaN layer, Al_0.12_Ga_0.88_N:Mn buffer and Al_0.27_Ga_0.73_N:Mn layers are indicated as 1, 2 and 3 respectively in the radial scans and RSM plots. The RSM and the radial 2*θ*-*ω* scan around the asymmetric (10
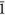
5) Bragg reflection of sample #D are reported in Fig. 3(a,b), where low, intermediate and high Q_*z*_ values correspond to the GaN layer, Al_0.12_Ga_0.88_N:Mn buffer and Al_0.27_Ga_0.73_N:Mn layers, respectively. The peaks of the three diffractions are aligned at the same value of Q_*x*_, pointing to a strained state of the films. The presence of higher-order superlattice or satellite peaks present in the RSM and 2*θ*-*ω* scan around the symmetric (0002) Bragg reflection of the same sample – as reported in Fig. 3(c,d), respectively – is a measure of the high crystallinity and of the periodicity of the layers. The Al content is quantified from the position of the (10
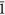
5) peak and according to the Vegard’s law satisfied by the considered compounds^45^. The obtained concentrations confirmed through energy-dispersive x-ray spectroscopy (EDX) measurements are similar for all the samples in the series, and correspond to (12.0 ± 1.0)% in the buffer and (26.9 ± 1.0)% in the Al_*x*_Ga_1−*x*_N:Mn Bragg layers, respectively. The Mn content is <0.2% cations both in the buffer and in the Bragg layers, as estimated from the EDX.

**Figure 4:**
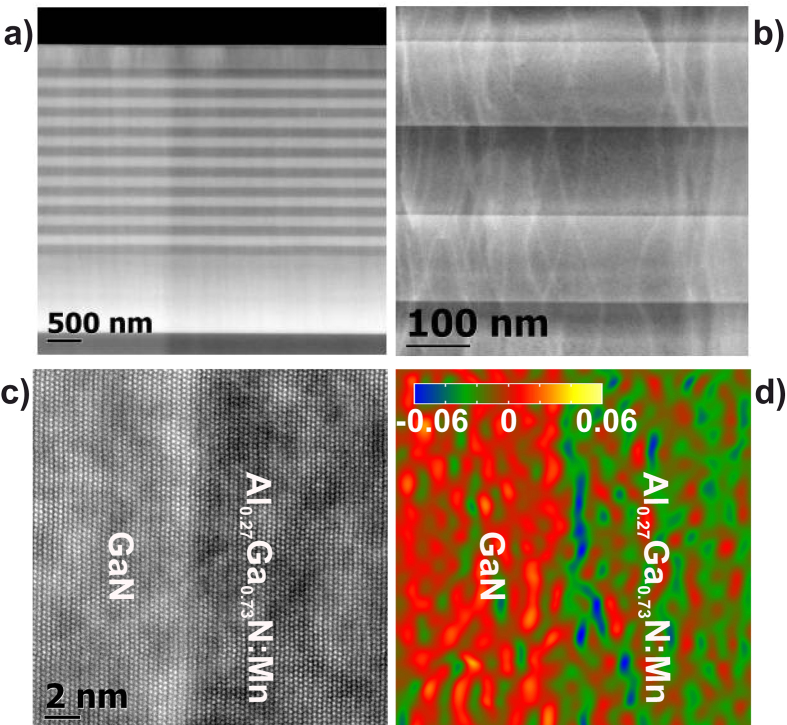
Panels (a,b) HAADF/STEM of sample #D. Light and dark alternating areas correspond to the GaN and Al_*x*_Ga_1−*x*_N:Mn regions of the Bragg pairs, respectively. In panel (a) the 130 nm thick GaN:(Mn,Mg) active layer is also distinguishable at the top of the structure. Panel (c) High resolution HAADF/STEM acquired along the 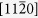 zone axis, with atomically defined interface between the GaN and Al_*x*_Ga_1−*x*_N:Mn layers of one Bragg pair. Panel (d) GPA strain mapping for the interface reported in panel (c).

The transmission electron microscopy (TEM) analysis of the structures points to the absence of major defects such as cracks or V-shaped ones in the heterostructures. Light and dark alternate regions in the high angle annular dark field (HAADF)/scanning TEM (STEM) image reported in Fig. 4(a,b) for sample #D with 10 pairs correspond to GaN and Al_*x*_Ga_1−*x*_N:Mn Bragg layers, respectively, while the defined Z-contrast in the HAADF/STEM image of Fig. 4(c) recorded on the [11
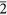
0] zone axis is an indication of the atomically sharp interface between the Al_*x*_Ga_1−*x*_N:Mn and the GaN layers. The HAADF/STEM has been recorded with a camera length of 145 mm for an acceptance angle 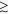
45 mrad of the detector. For this high acceptance angle, the HAADF detector can also detect the diffraction patterns from higher order Laue zones (HOLZ), which are sensitive to the structural characteristics like defects and dislocations and can appear as curve linear contrast in the HAADF image^49^. The HOLZ line contrast of threading dislocations for sample #D can be seen in Fig. 4(b). The thickness of the single layers is in accord with the nominal one expected from the growth parameters and with those required by the TMM model.

To study the threading dislocations in the DBR samples #B, #D and #F, weak-beam-dark-field (WBDF) images have been recorded under 1 g/3 G weak-beam conditions with g = 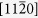, where g is parallel to the Burger’s vectors of edge dislocation. The dislocation densities in the samples have been estimated using cross sectional specimens and are found to be of the order of (5.8 ± 0.6) × 10^10^ cm^−2^ for the Al_*x*_Ga_1−*x*_N:Mn buffer, while for the DBR region reduce to (1.9 ± 0.3) × 10^10^ cm^−2^. These quantitative values are comparable for all the DBR samples irrespective of the periods of the DBR superlattice. The observed dislocations are dominated by defects due to structural and thermal mismatch of the Al_*x*_Ga_1−*x*_N nucleation layer with respect to the sapphire substrate, while the reduction in the dislocation density in the DBR is due to recombination of the edge dislocations in regions around the first DBR layer adjacent to the buffer layer. The strain state around the Al_*x*_Ga_1−*x*_N:Mn/GaN interface already evidenced by x-ray diffraction (XRD) is confirmed by the geometric phase analysis (GPA) reported in Fig. 4(d). In the studied area, the average out-of-plane strain of the Al_*x*_Ga_1−*x*_N:Mn layer with respect to the GaN layer is *ε*_⊥_ = −0.012, while the in-plane strain *ε*_‖_ – figure not shown – is random with a statistical average of 0, which indicates a pseudomorphic growth of Bragg pairs on the Al_0.12_Ga_0.88_N:Mn buffer.

### Effect of the Al_*x*_Ga_1−*x*_N:Mn/GaN DBR on the optical response of the active layer

**Figure 5:**
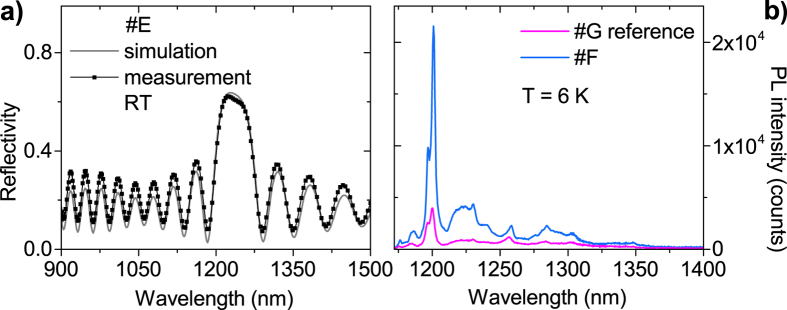
Left panel (a) Room temperature - measured and calculated - reflectivity of the sample #E with 20 Bragg pairs confirming the agreement between measurement and simulation. Right panel (b) Low temperature PL comparing the sample #F with the reference sample #G. A significant enhancement of the PL signal is observed.

The simulations performed by applying the TMM method, prove to be a powerful tool to predict the effect of the Bragg layer and Bragg pair thickness on the position of the stop-band of the DBR as well as to estimate the maximum reflectivity signal. The measured reflectivity spectra are in agreement with the simulations carried out for the multilayer structures – as evidenced in the Fig. 5(a) for sample #E with 20 Bragg pairs. The accordance between measurements and modeling confirms that in the perspective of light extraction Al_*x*_Ga_1−*x*_N(:Mn)/GaN-based DBRs are quite insensitive to a dislocation density in the DBR as high as 1.9 × 10^10^ cm^−2^ as also reported by Nakamura *et al*.^50^ for blue LEDs.

The effect of the DBR on the photoluminescence (PL) signal is highlighted in Fig. 5(b) for sample #F, which has the same architecture as sample #E with a stop-band around 1200 nm, but with the addition of the GaN:(Mn,Mg) active layer. For the reflectivity value of 62%, the Mn-Mg_*k*_-related PL intensity at 1200 nm is already at least five times greater that the one from sample #G, *i*.*e*. a GaN:(Mn,Mg) active layer without DBR.

As mentioned in the Methods, the measurements of reflectivity are carried out at room temperature. By considering the changes in the band-gap as a function of temperature for Al_*x*_Ga_1−*x*_N(:Mn) and GaN^51^, at 6 K a ~5 nm shift of the DBR stop-band center for the 137 nm/131 nm Al_*x*_Ga_1−*x*_N(:Mn)/GaN Bragg pairs is expected, with a consequent increase of the reflectance at 1200 nm. A similar effect has been reported from PL and reflectivity on Al_*x*_Ga_1−*x*_N/Al_*y*_Ga_1−*y*_N DBRs for the UV range^30^. We have studied also the effect of the Al_0.12_Ga_0.88_N:Mn buffer layer thickness on the reflectance and the simulations are available as video in the Supplementary Information. The full set of PL measurements at 6 K for samples #B, #D, #F and for the reference #G is reported in Supplementary Fig. S4.

### Modeling and strain analysis

As reported in Fig. 5(a), the reflectivity of the investigated structures is ~62%. According to the simulations, by increasing to *x* ~ 60% the Al concentration in the low refractive index layers the reflectivity is expected to increase to ~95%, and an increment in the number of Bragg pairs from 20 to 30 would further enhance the reflectivity to ~99%. The main challenge in growing such structures with a high Al content is the critical thickness of Al_*x*_Ga_1−*x*_N on GaN, limited by stress accumulation. The strain energy in the DBR layer due to the lattice mismatch can be estimated as 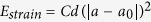, where *C* is the elastic constant, *d* the layer thickness, *a*_0_ and *a* the in-plane lattice parameters of DBR and buffer layer, respectively. According to this estimation, the minimum of the total strain energy is obtained for the Al_*y*_Ga_1−*y*_N buffer layer with a value of the in-plane lattice parameter which is intermediate between the one of GaN and the one of Al_*x*_Ga_1−*x*_N^28^. In this way, the compressive stress in the GaN layer and the tensile stress in the Al_*x*_Ga_1−*x*_N one compensate each other and pseudomorphic growth of a DBR structure is possible. To estimate the total strain energy in the DBR, a linear interpolation of the elastic constants of GaN and AlN can be employed for Al_*x*_Ga_1−*x*_N at different Al concentration, since the values *C*_11_, *C*_12_ for GaN and AlN do not differ significantly, being *C*_11_ = 390 and *C*_12_ = 145 GPa for GaN and *C*_11_ = 410 and *C*_12_ = 149 GPa for AlN, respectively^52^. As a result, by incrementing the Al concentration from 30% to 60% and augmenting the number of Bragg pairs from 20 to 30, the increase of the total strain energy in the structure is enhanced by a factor ~6.4.

## Discussion

All-nitride Al_*x*_Ga_1−*x*_N:Mn/GaN based DBR structures for the NIR range have been designed, fabricated and tested in combination with GaN:(Mn,Mg) layers optically active in the near-infrared range of wavelengths. Simulations based on the TMM method provide an indispensable tool to design and tune the thickness of the various layers constituting the investigated heterostructures. Photoluminescence measurements up to room temperature reveal the enhancement of the emission intensity from Mn-Mg_*k*_ complexes in a GaN:(Mn,Mg) layer grown on the DBR structure, opening up concrete perspectives for the realization of a NIR nitride-based laser. As the technology for quantum light sources evolves, the development of single photon emitters becomes an essential stage on the roadmap of nitride-based devices^53^,^54^. The zero-dimensional nature of the Mn-Mg_*k*_ cation complexes – which identifies them as solotronic objects – in GaN:(Mn,Mg), together with their structural stability and in combination with tunable Al_*x*_Ga_1−*x*_N:Mn/GaN DBRs paves the way for the design and fabrication of nitride-based single-photon sources^55^.

## Methods

### Modeling

The design of the DBRs in this work is supported by reflectivity simulations based on the TMM^46^. With this formalism, the relation between the electric fields of the incident, reflected and transmitted light is given by modeling the multilayer structure as a series of interfaces and propagation regions represented by a scattering matrix (system transfer matrix), which is the successive product of: (i) the refractive matrices describing the reflection and transmission at a single interface and (ii) the phase matrices accounting for the phase shift caused by the propagation through a layer. Within this model, the whole transmission and reflectance spectrum of an arrangement of dielectric layers can be obtained, once the refractive indices of the involved materials are known. For the present work, the refractive indices of Al_*x*_Ga_1−*x*_N:Mn alloys with different Al content and 0.2% of Mn, have been established by spectroscopic ellipsometry measurements. Details on the samples specifically fabricated for the refractive index studies, as well as on the *ex situ* ellipsometry measurements, are provided in the Supplementary Information.

### Epitaxial growth

All the samples are grown by MOVPE on 2”*c*-plane sapphire substrates in an AIXTRON 200RF horizontal reactor, according to procedures we have described elsewhere^16^,^45^,^56^. The precursors employed for Ga, N, Al, Mn and Mg are trimethylgallium (TMGa), ammonia (NH_3_), trimethylaluminium (TMAl), bis-methylcyclopentadienyl-manganese (MeCp_2_Mn), and dicyclopentadienyl-magnesium (Cp_2_Mg) respectively. For all processes the flow-rate of NH_3_ is kept at 1500 standard cubic centimeters per minute (sccm), while all the other flow-rates, together with reactor pressures and temperatures are reported in the Supplementary Table S1. The deposition process is carried out under H_2_ atmosphere. After the growth of a Al_*x*_Ga_1−*x*_N nucleation layer at 540 °C and *p* = 200 mbar, the annealing process is carried out at 975 °C. A 1 *μ*m Al_0.12_Ga_0.88_N:Mn buffer layer is then deposited epitaxially at 975 °C and *p* = 100 mbar. Upon deposition of the buffer, the Al_0.27_Ga_0.73_N:Mn/GaN Bragg pairs are grown at the same temperature, and in samples #B, #D, and #F, a 130 nm thick GaN:(Mn,Mg) active layer is deposited at 850 °C and *p* = 200 mbar. For sample #G the GaN:(Mn,Mg) layer deposition starts directly after the Al_0.12_Ga_0.88_N:Mn buffer, providing a reference without Bragg reflector.

### Characterization

*In situ* and on-line kinetic ellipsometry ensures the direct control of the deposition process and provides information on the thickness of the layers, which is then confirmed by *ex situ* spectroscopic ellipsometry and TEM in both conventional (CTEM) and scanning mode (STEM), performed in a FEI Titan Cube 80–300 operating at 300 keV and in a JEOL 2010 F working at 200 keV. Bright/dark-field (BF/DF), high resolution TEM (HRTEM) and high angle annular dark field (HAADF) are employed for the in-depth structural characterization of the structures, and mapping is performed with energy filtered TEM (EFTEM), at the Al *K* absorption edge. Cross-section TEM specimens are prepared by mechanical polishing, dimpling and final ion milling in a Gatan Precision Ion Polishing System.

Information on the morphology of the surface is obtained from atomic force microscopy (AFM) in tapping mode with a VEECO Dimension 3100, while the Al concentration is calculated from the position of the (0002) and (10
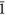
5) diffraction peaks of Al_*x*_Ga_1−*x*_N(:Mn), measured on a PANalytical’s X’Pert PRO Materials Research Diffractometer (MRD) equipped with a hybrid monochromator with a 1/4° divergence slit. The diffracted beam is measured with both a triple axis and a solid-state PixCel detector used as 256-channels detector with a 9.1 mm anti-scatter slit. Reflectivity measurements are carried out at room temperature with a Bruker VERTEX 80 Fourier-transform IR spectrometer. PL spectra are acquired at 6 K and at room temperature, using a diode laser with an excitation wavelength of 442 nm and an InGaAs line detector.

## Additional Information

**How to cite this article:** Capuzzo, G. *et al*. All-nitride Al_*x*_Ga_1-*x*_N:Mn/GaN distributed Bragg reflectors for the near-infrared. *Sci. Rep.* **7**, 42697; doi: 10.1038/srep42697 (2017).

**Publisher's note:** Springer Nature remains neutral with regard to jurisdictional claims in published maps and institutional affiliations.

## Supplementary Material

Supplementary Information

Supplementary Video

## Figures and Tables

**Table 1:** Investigated samples: number *n* of Bragg pairs and presence of GaN:(Mn,Mg) active layer.

Sample	Number *n* of Bragg pairs 137 nm/131 nm (Al_0.27_Ga_0.73_N:Mn/GaN)	GaN:(Mn,Mg) active layer (130 nm)
#A	5	no
#B	5	GaN:(Mn,Mg)
#C	10	no
#D	10	GaN:(Mn,Mg)
#E	20	no
#F	20	GaN:(Mn,Mg)
#G	0	GaN:(Mn,Mg)

In samples #B, #D and #F, the 1200 nm emission from the Mn-Mg_*k*_ complexes in the GaN:(Mn,Mg) active layer is in the range of maximum reflectivity of the DBR (stop-band).
