# Supplementary material for: All-nitride AlxGa1−xN:Mn/GaN distributed Bragg reflectors for the near-infrared
Source: Sci Rep. 2017 Feb 15;7:42697. doi: 10.1038/srep42697 (PMC5309887; doi:10.1038/srep42697)
Supplement: Supplementary Information [file srep42697-s1.pdf]

All-nitride $Al_xGa_{1-x}N$:Mn/GaN distributed Bragg reflectors for the near-infrared

Giulia Capuzzo,[1,*] Dmytro Kysylychyn,[1] Rajdeep Adhikari,[1] Tian Li,[2]
Bogdan Faina,[1] Aitana Tarazaga Martín-Luengo,[1] and Alberta Bonanni[1,†]

[1]*Institut für Halbleiter-und-Festkörperphysik,*
Johannes Kepler University, Altenbergerstr. 69, A-4040 Linz, Austria

[2]*Institute of Physics, Polish Academy of Science,*
al. Lotnikow 32/46, 02-668 Warsaw, Poland

Growth parameters of the DBR structures

The growth parameters employed during the metalorganic vapor phase epitaxy (MOVPE) of the various layers constituting the $Al_xGa_{1-x}N$:Mn/GaN-based samples are summarized in Table S1, together with those of the GaN:(Mn,Mg) active layers.

Table S1. Growth parameters employed during the MOVPE process for the fabrication of the different epilayers/heterojunctions necessary for the architecture of the full DBR structure, eventually completed with a GaN:(Mn,Mg) active layer. The flow rates of the precursors are given in standard cubic centimeters per minute (sccm).

Layer	TMGa (sccm)	TMAl (sccm)	$MeCp_2Mn$ (sccm)	Cp_2Mg (sccm)	Reactor pressure (mbar)	Reactor temperature (°C)
$Al_xGa_{1-x}N$ NL	10	10	–	–	200	540
$Al_{0.12}Ga_{0.88}N$:Mn buffer	10	30	50	–	100	975
$Al_{0.27}Ga_{0.73}N$:Mn (DBR)	5	60	50	–	100	975
GaN (DBR)	25	–	–	–	100	975
GaN:(Mn,Mg)	5	–	75	450	200	850

A list of a complete series of samples grown for this work is reported in Table S2. Samples #A - #F have a stop-band centered at around 1200 nm, while samples #G and #H - at 980 nm, and sample #I is a reference sample containing an active GaN:(Mn,Mg) layer grown directly on the $Al_{0.12}Ga_{0.88}N$:Mn buffer.

Ex situ **ellipsometry measurements to establish the value of the refractive indices**

The refractive indices of $Al_xGa_{1-x}N$:Mn alloys with different Al content and 0.2% of Mn are reported in Fig. S1 together with those extrapolated from the model based on the first-order Sellmeier dispersion formula employed by Özgür *et al.* [1] for $Al_xGa_{1-x}N$. The values are shown for wavelengths between 900 nm and 1500 nm, corresponding to the range of NIR emission from the Mn-Mg_k complexes in the GaN:(Mn,Mg) active layer of specific interest[2].

The refractive indices of the $Al_xGa_{1-x}N$:Mn alloys with different Al content have been studied with a spectroscopic M-2000 ellipsometer, J.A. Woollam Co.. The ellipsometer is capable of measurements in the energy range between 0.73 eV and 6.5 eV (190 nm-1600 nm).

Table S2. Investigated samples: number n of Bragg pairs and presence of GaN:(Mn,Mg) active layer. In samples #B, #D and #F, the 1200 nm emission from the Mn-Mg$_k$ complexes in the GaN:(Mn,Mg) active layer is in the range of maximum reflectivity of the DBR (stop-band).

Sample	Number n of Bragg pairs 137 nm/131 nm ($Al_{0.27}Ga_{0.73}N$:Mn/GaN)	GaN:(Mn,Mg) active layer (130 nm)
#A	5	no
#B	5	GaN:(Mn,Mg)
#C	10	no
#D	10	GaN:(Mn,Mg)
#E	20	no
#F	20	GaN:(Mn,Mg)
#G	20 (112 nm/112 nm)	no
#H	20 (112 nm/112 nm)	GaN:(Mn,Mg)
#I	0	GaN:(Mn,Mg)

It is equipped with a deuterium lamp for the UV-to-visible region and with a quartz-tungsten halogen (QTH) lamp for the visible-to-IR region. A rotating-compensator configuration combined with a CCD-detector is employed to carry out the simultaneous measurements of 500 wavelengths. The sample holder is provided with a vacuum pump to fix the sample and it can be tilt-adjusted in the x- and y-directions with $2\,\mu$m screws. The measurements are performed at different angles of incident light between 55° and 75°, at a 5° step to improve the accuracy of the fitting.

The fitting procedure relies on the Software Complete Ease, J.A. Woollam Co., Inc., Version 5.04. Depending on the optical response of the material in the measured energy (wavelength) range, each layer can be fitted with appropriate dispersion models. If the material under consideration is transparent or partially transparent in the measured energy range, then the Cauchy dispersion law with an Urbach absorption tail can be employed: since the spectral region of interest in the present work lies in the infrared, where the GaN-based materials are transparent, the Cauchy model is utilized. In this case, three terms describe the refractive

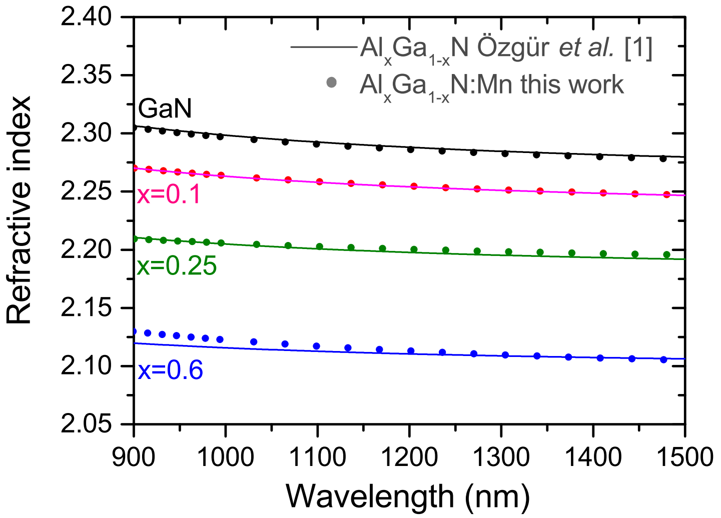

Figure S1. Refractive indices of $Al_xGa_{1-x}N$:Mn with various Al contents and 0.2% Mn (dots) compared with those reported by Özgür *et al.* [1] for $Al_xGa_{1-x}N$ (solid lines), extrapolated for NIR wavelengths.

index and an exponentially decaying function accounts for the extinction coefficient:

$$n(\lambda) = A + \frac{B}{\lambda^2} + \frac{C}{\lambda^4} \tag{1}$$

$$k(\lambda) = \alpha e^{\beta\left(1.24\mu m(\frac{1}{\lambda}-\frac{1}{\gamma})\right)} \tag{2}$$

where the A, B, C parameters are variable fit parameters that determine the index of dispersion; α is the extinction coefficient amplitude, β the exponent factor, γ the band-edge, with λ and γ in μm. The sapphire substrate on which the samples studied in the present work are epitaxially grown, can be fitted with a Cauchy model in the whole measured energy range.

The samples that have been used to determine the refractive index of GaN and $Al_xGa_{1-x}N$:Mn at different Al concentration – summarized in Table S3 – consist of: a 1 μm GaN buffer layer deposited epitaxially by MOVPE after the growth of a low temperature GaN nucleation layer (sample S1), a 5-period $Al_{0.25}Ga_{0.75}N$:Mn/GaN DBR grown on top of a 1 μm $Al_{0.1}Ga_{0.9}N$:Mn buffer (sample S2) and a 1 μm $Al_{0.6}Ga_{0.4}N$:Mn buffer deposited after the growth of a low temperature $Al_xGa_{1-x}N$ nucleation layer (sample S3), according to the model sketched in Fig. S2. The thicknesses of the Bragg pairs in sample S2 are 130 nm and

165 nm for the GaN and $Al_{0.25}Ga_{0.75}N$:Mn layers, respectively. Sample S1 is employed to determine the refractive index of GaN, sample S2 for the refractive index of $Al_xGa_{1-x}N$:Mn with x=0.10 and x=0.25, and sample S3 for the one of $Al_{0.6}Ga_{0.4}N$:Mn.

Table S3. Samples used for ellipsometry studies to determine a refractive index of $Al_xGa_{1-x}N$(:Mn) at different Al content.

Sample	Buffer	Layers	Determined refractive index
S1	GaN	–	GaN
S2	$Al_{0.1}Ga_{0.9}N$:Mn	5x($Al_{0.25}Ga_{0.75}N$:Mn/GaN)	$Al_{0.1}Ga_{0.9}N$:Mn, $Al_{0.25}Ga_{0.75}N$:Mn
S3	$Al_{0.6}Ga_{0.4}N$:Mn	–	$Al_{0.6}Ga_{0.4}N$:Mn

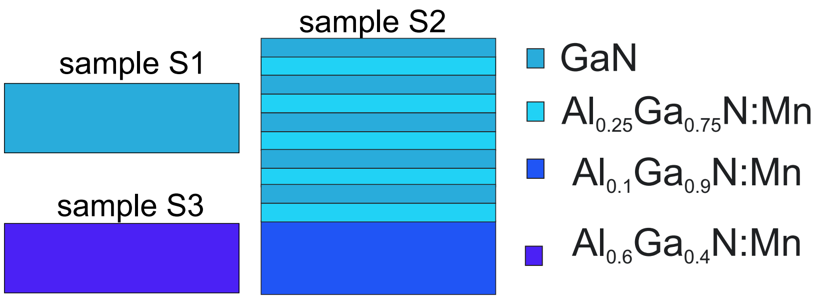

Figure S2. Architecture of the dedicated heterostructures grown to establish the refractive index of GaN and $Al_xGa_{1-x}N$:Mn with different Al concentrations. All samples are deposited on c(0001)-sapphire substrates.

Reflectivity simulations and stop-band tuning

The position of the stop-band in the reflectivity spectra can be finely tuned by changing the thicknesses of the $Al_{0.27}Ga_{0.73}N$:Mn and GaN films, *e.g.* a decrease in the thickness (of the single Bragg layers and, consequently, of the Bragg pair) shifts the center of the stop-band towards low wavelengths. For example – as evidenced in Fig. S3 a) – for sample #G, which consists of 20 Bragg pairs, each of them having 112 nm-thick $Al_{0.27}Ga_{0.73}N$:Mn and GaN layers, the center of the stop-band is at 980 nm. On the other hand, an increment of the $Al_{0.27}Ga_{0.73}N$:Mn and GaN layers thickness - as in sample #E - shifts the center of

the stop-band to 1235 nm, as seen in Fig. S3 b). The measured reflectivity spectra are in agreement with the simulations carried out for the multilayer structure with the thicknesses discussed above.

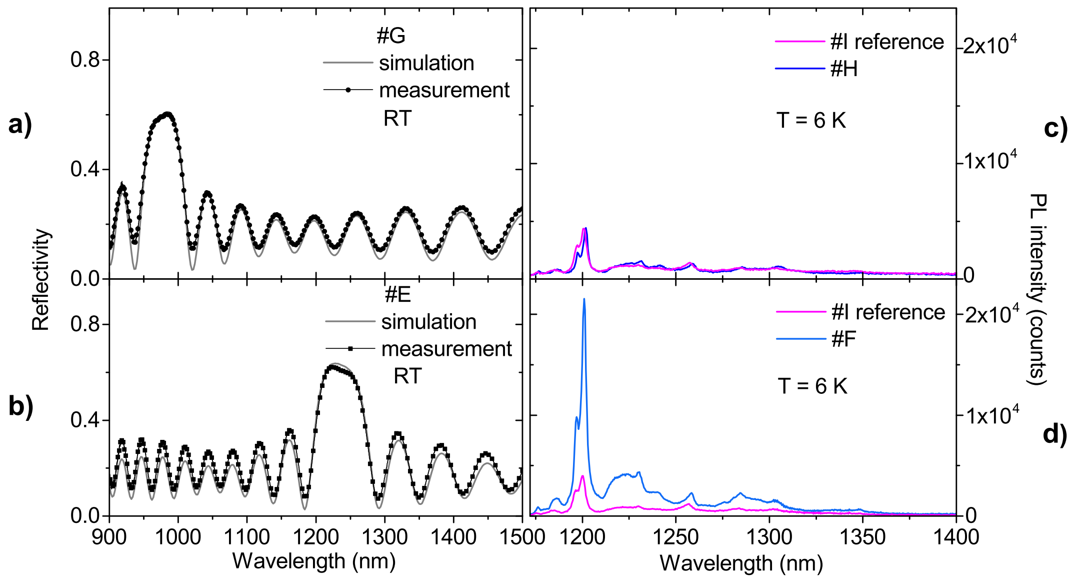

Figure S3. Left panels: room temperature - measured and calculated - reflectivity of samples #G a) and #E b) with 20 Bragg pairs confirming the tunability of the stop-band with respect to the Bragg layers thickness. Right panels: low temperature PL comparing sample #H - c) and #F - d) with the reference sample #I. A significant enhancement of the PL signal when the stop-band is closer to 1200 nm is observed.

The effect of shifting the stop-band affects the photoluminescence (PL) signal around 1200 nm for the samples having a GaN:(Mn,Mg) active layer on top of a DBR structure. For example, the DBR in samples #G and #H generates a stop-band centered at 980 nm, as previously discussed, and consequently, no enhancement in the around-1200 nm PL intensity (no effect of the Bragg reflector) is observed for sample #H (with GaN:(Mn,Mg) active layer) compared to sample #I (reference GaN:(Mn,Mg) active layer without DBR), as evidenced in Fig. S3 c). In contrast, sample #F (with GaN:(Mn,Mg) active layer), having a DBR with stop-band centered around 1200 nm, shows a Mn-Mg_k-related PL recombination at 1200 nm which is at least five times greater that the one from sample #I (reference GaN:(Mn,Mg) active layer without DBR), as highlighted in Fig. S3 d).

Reflectivity simulations of DBRs *vs* buffer thickness

The effect of an $Al_{0.12}Ga_{0.88}N$:Mn buffer layer on the $Al_{0.27}Ga_{0.73}N$:Mn/GaN DBR reflectance has been studied *via* the transfer matrix method (TMM) for a structure containing 20-Bragg pairs. When the condition described by Eq. 3 - where n and d are respectively the refractive index and the thickness of the considered layer, while λ is the design wavelength - is fulfilled for odd values of k, then the reflectance reaches a maximum. Even values of k correspond to a minimum of the reflectance.

$$nd = k\frac{\lambda}{2}, k\epsilon\mathbb{N} \tag{3}$$

In our case, the design wavelength is 1200 nm and the simulation starts from the nominal thickness at which the $Al_{0.12}Ga_{0.88}N$:Mn is grown, namely 1μm, that corresponds to a total reflectance of 60%. As highlighted in the video, the minimum of the reflectance is met at 1072 m, while the condition for maximum reflectance is fulfilled at 1206 nm, bringing the reflectance to an increase of 10% with respect to the initial value.

Video legend: Effect of the $Al_{0.12}Ga_{0.88}N$(:Mn) buffer layer thickness on the reflectance simulated for the sample with 20-fold 137/131 nm $Al_{0.27}Ga_{0.73}N$(:Mn)/GaN DBR.

Photoluminescence

The full set of PL measurements at 6 K for samples #B, #D, #F and for the reference #I is reported in Fig. S4. As expected, the intensity of the main Mn-Mg_k-related peak at 1200 nm increases as a function of the number of pairs in the DBR. By comparing the PL intensity of reference sample #I with the one of sample #F, where the 20-fold DBR has been added, there is an increment of about 5.4 times in the measured intensity. This effect persists up to room temperature, where the intensity of the PL spectra is lower but preserves a systematic dependence on the number of Bragg pairs in the DBR, as reported in the inset to Fig. S4.

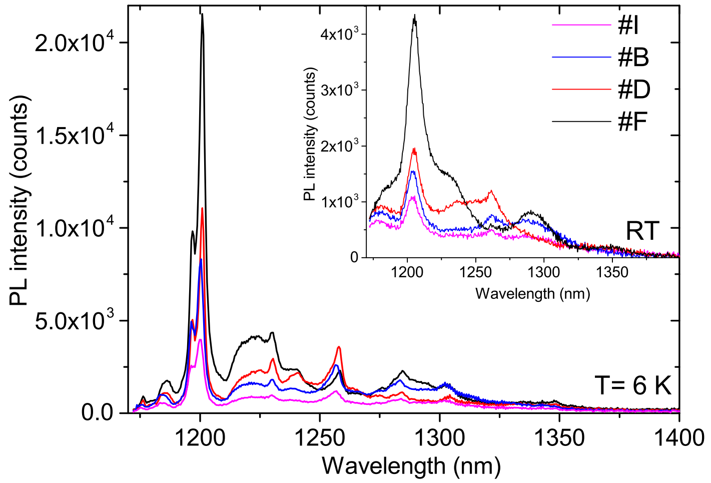

Figure S4. PL measurements for samples #B (5 Bragg pairs and active layer), #D (10 Bragg pairs and active layer), #F (20 Bragg pairs and active layer) and for the reference #I (active layer without Bragg pairs) at T = 6 K and at room temperature (inset).

---

* giulia.capuzzo@jku.at

† alberta.bonanni@jku.at

1 Özgür, Ü., Webb-Wood, G., Everitt, H. O., Yun, F. & Morkoç, H. Systematic measurement of $Al_xGa_{1-x}N$ refractive indices. *Appl. Phys. Lett.* **79**, 4103–4105 (2001).

2 Devillers, T. *et al.* Manipulating Mn-Mg_k cation complexes to control the charge- and spin-state of Mn in GaN. *Sci. Rep.* **2**, 722 (2012).
